# Practical Notes and Formulæ

**Published:** 1886-09-20

**Authors:** 


### PRACTICAL NOTES AND FORMULÆ.

DR. ARTHUR V. MEIGS, in the *Phildelphia Medical Journal*, recommends the following formula for food for infants:

| | | |
|---|---|---|
| ℞. | Cream | 2 tablespoonfuls, |
| | Milk (cow's) | 1 tablespoonfuls, |
| | Lime-water | 2 tablespoonfuls, |
| | Sugar-water | 3 tablespoonfuls. |

The sugar-water is made by dissolving 17¾ drachms of sugar of milk in a pint of water. The above mixture more nearly resembles in composition human milk than any other he knows of. In case of partial failure to digest the casein, resulting in fœcal accumulation, he substitutes 3 tablespoonfuls of water and a level teaspoonful of Mellin's food. He objects to the use of condensed milk, but admits he has seen it answer a good purpose in infant-feeding.—*North Carolina Medical Journal.*

THE following is a formula given by Dr. Levis, much used at the Pennsylvania Hospital for *fixed dressing:*

| | | |
|---|---|---|
| ℞. | Glue | 1 pound, |
| | Oxide of zinc | 2 pounds, |
| | Water | Cong. 1. |

M. Sig.—To be applied while hot, by means of a brush.

It takes only a few hours to harden, in this respect being preferable to the silicate of soda dressing.—*Col. and Clin. Record.*

**For Vaginismus.**—In cases of vaginal inflammation, accompanied with more or less uterine tenderness and leucorrhœa:

| | | |
|---|---|---|
| ℞. | Fluid golden-seal, colorless, } Distilled ext. hammamelis, } aa | ℥ j, |
| | Aqueos ext. pinus canadensis | ℥ ss, |
| | Water | ℥ jss. |

M. Saturate a small wad of antiseptic cotton with this, and through a speculum place it in the vagina. Applications of this kind are very effective.—*American Medical Journal.*

**Cough.**—

| | | |
|---|---|---|
| ℞. | Aluminis | gr. x, |
| | Aq | fʒ j. |

As spray to throat.

| | | |
|---|---|---|
| ℞. | Croton chloral hydrat | gr. xxxvj, |
| | Glycerini | ʒ iij, |
| | Aq. dest. ad | f℥ vj. |

M. S.—Two tablespoonfuls every five hours.

℞. Tannici glycerini, as paint to throat.

℞. Moph. sulphat .......... gr. ss,

Quinæ sulphat .......... ʒ j,

Acid. hydrochlor. dil .......... fʒ vj.

M. S.—One teaspoonful in water, four times a day.—*Medical World.*

**For Intermittent Fever.**—Dr. A. H. Merry, writing to *New England Medical Monthly*, says: I have found the following formula very useful in breaking up chills and fever, one lot of the pills being generally sufficient.

℞. Quiniæ sulph .......... grs. xvi,
Calomel .......... grs. viij,
Resin of podophyllin .......... gr. ss,
Gelsemin .......... gr. jss.

Mix and make into eight pills. Sig. Take one every hour till all are taken. They want to be taken after the fever has gone.—*American Medical Digest.*

The following formula is highly recommended by Dr. Duffy in the treatment of subacute rheumatism with anæmia, or the anæmia following acute rheumatism:

℞. Acid salicylic .......... grs. viii,
Sodii salicylat .......... grs. iv,
Ferri malat .......... gr. i,
Aqua .......... ʒ ij.
M. Sig.—One dose; to be taken every four hours.—*Buffalo Medical and Surgical Journal.*

**For Rheumatism.**—A. J. Conger has personally tested the following, and commends it:

℞. Potass. iodidi .......... ℥ ijss,

Tr. cimicifugæ .......... ℥ iss,

Vin. colchici sem .......... ℥ i,

Ext. hyosciami fl .......... ℥ ss,

Syr. simplici .......... ℥ v

M. Sig.—Teaspoonful well diluted every four hours.—*N. E. Medical Monthly.*

**For Gonorrhœa.**—Dr. J. H. Stanley, in *Medical World*, gives the following:

℞. Balsam copaibæ .......... ℥ iij,

Spt. nit. dulc .......... ℥ j,

Spt. lavender .......... ℥ j,

Olei terbinth .......... ʒ j.

M. S. Take one teaspoonful twice a day.

For injection:

℞. Plumbi acetatis .......... gr. xl,
Zinc sulphatis .......... gr. xl,
Pulv. acaciæ .......... gr. xl,
Tinct. opii .......... ℥ j.
Aquæ dest .......... O. J.
M. S. Inject morning and evening.

